# Reproductive Regulation and Oxidative Stress Alleviation of Chinese Herbal Medicine Therapy in Ovariectomised Mouse Model

**DOI:** 10.1155/2019/5346518

**Published:** 2019-07-07

**Authors:** Chung-Hsin Wu, Sheue-Er Wang, Chih-Hsiang Hsu, Yu-Tsen Hsu, Chen-Wen Lu, Wu-Chang Chuang, Ming-Chung Lee

**Affiliations:** ^1^School of Life Science, National Taiwan Normal University, Taipei City, Taiwan; ^2^Pathological Department, Saint Paul's Hospital, Taoyuan City, Taiwan; ^3^Sun Ten Pharmaceutical Co. Ltd., New Taipei City 23143, Taiwan; ^4^Brion Research Institute of Taiwan, New Taipei City 23143, Taiwan

## Abstract

In Taiwan, the herbal formula B401 is considered as a health supplement for middle-aged women that can alleviate sweating, anxiety, and sleep disorders. However, the relevant mechanisms are still unclear. In this study, we evaluated the beneficial effects of the herbal formula B401 therapy in the reproductive regulation of ovariectomised mice. Female ICR mice were randomised into four groups: wild-type (WT) mice with sham treatment, wild-type mice treated with the herbal formula B401, bilateral ovariectomised (OVX) mice with sham treatment, and bilateral ovariectomised mice treated with the herbal formula B401. Mice were orally given the herbal formula B401 at a dose of 30 mg/kg bw/day for 2 weeks. At the end of oral treatment with sham or the herbal formula B401, levels of reactive oxygen species (ROS), calcium, phosphorus, and estradiol-17*β* in the blood; uterine weight and endometrial thickness; and expressions of estrogen receptor *α* (ER*α*), estrogen receptor *β* (ER*β*), progesterone receptor (PR), vascular endothelial growth factor (VEGF), and superoxide dismutase 2 (SOD2) in the uterine tissue were examined and then compared among the four groups of mice. We found that OVX mice decreased levels of calcium, phosphorus, and estradiol-17*β* in the blood, decreased uterine weight and endometrial thickness, and decreased expressions of ER*α*, ER*β*, PR, and SOD2 in the uterine tissue but increased blood ROS levels compared with those of WT mice. In addition, OVX mice with the herbal formula B401 therapy can increase levels of calcium, phosphorus, and estradiol-17*β* in the blood, increase uterine weight and endometrial thickness, and increase expressions of ER*α*, ER*β*, PR, VEGF, and SOD2 in the uterine tissue but decrease blood ROS levels. Our results may provide reasonable explanation for the reproductive regulation of the herbal formula B401 therapy.

## 1. Introduction

Some menopausal women experience discomfort symptoms such as hot flashes, sweating, anxiety, sleep disorders, and osteoporosis [1]. These uncomfortable symptoms are usually caused by the reduction or stoppage of ovarian estrogen production. Estrogen is the primary female sex hormone and plays multiple biological functions, including bone remodelling and neuroprotection through the modulation of the estrogen receptors: estrogen receptor *α* (ER*α*) and estrogen receptor *β* (Er*β*) [1, 2]. Estrogen therapy is currently the most common treatment for alleviating menopausal symptoms [3]. Estrogen may affect bone remodelling by accelerating calcium and phosphorus reabsorption by the intestine and reducing calcium and phosphorus excretion from the kidney [4]. Furthermore, estrogen can reduce oxidative stress involved in the pathogenesis of the reproductive regulation. A previous study reported that ovariectomised (OVX) rats showed increased levels of reactive oxygen species (ROS) and reduced expression of antioxidant enzymes such as superoxide dismutase 2 (SOD2) [5]. Menopausal symptoms are often caused by mitochondrial oxidative stress because of the decline of the natural antioxidant estrogen [6].

Even though estrogen therapy is considered effective for alleviating menopausal symptoms, it is associated with high risks of cardiovascular disease, endometrial hyperplasia, and breast cancer [7]. Thus, many women refuse to undergo exogenous estrogen hormone therapy and switch to alternative treatment for alleviating menopausal symptoms. In Taiwan, for menopausal symptom remission, alternative medicines such as traditional Chinese medicines are becoming popular because of their reputed safety and clinical efficacy. In traditional Chinese medicine theory, menopausal symptoms are often triggered by kidney–liver weakness [8]. Herbal formulas classified as kidney/liver-nourishing substances should be suitable for menopausal symptom remission. It is believed that complex interactions of components in herbal formulas can produce synergistic effects, and these herbal formulas show reduced side effects and toxicity. Thus, in the present study, we adopted a modified herbal formula B401 for reproductive regulation in ovariectomised mice. The herbal formula B401, consisting of* Angelica sinensis*,* Astragalus membranaceus*,* Eclipta prostrata*,* Ligustri fructus*,* Panax ginseng*, and* Rehmannia glutinosa*, is widely used in Taiwan and is considered as a health supplement that supports healthy brain and reproductive functions. Our previous studies have reported that the herbal formula B401 can suppress oxidative stress, inflammation, and apoptosis in the brain and reproductive systems of mice [9–13]. Recently, the herbal formula B401 is also used as a health supplement for middle-aged women in sweating, anxiety, and sleep disorders alleviation. However, the relevant mechanisms regarding the herbal formula B401 therapy alleviating discomfort symptoms are still unclear. Thus, in this study, we evaluated the beneficial effects of the herbal formula B401 on reproductive regulation in ovariectomised mice. We hope that our results may provide reasonable explanation for the reproductive regulation of the herbal formula B401 therapy.

## 2. Materials and Methods

### 2.1. Preparation of the Herbal Formula B401

The main components of the herbal formula B401 (US patent, No 7838048B2) are* Ang. sinensis*,* Ast. membranaceus*,* E. prostrata*,* L. fructus*,* P. ginseng*, and* R. glutinosa *in specific ratios. The chromatographic fingerprint of the herbal formula B401 was assayed on a liquid chromatography/mass spectrometry (LC/MS) analytical system equipped with a LC-20AD UFLC system (Shimadzu Corporation, Kanagawa, Japan) linked with a LCMS-8040 triple mass spectrometer (Shimadzu Corporation).

### 2.2. Cytotoxicity Assay

SH-SY5Y cells were treated with and without (control) the herbal formula B401 at indicated doses for 24 h to determine the IC_50_ of cytotoxicity. Subsequently, cell viability was measured through the 3-(4,5-dimethylthiazol-2-yl)-2,5-diphenyltetrazolium bromide (MTT) assay (Sigma-Aldrich Corporation, St. Louis, MO, USA). Absorbance was measured at OD 570 nm on an ELISA reader (uQuant, BioTek Inc., Vermont, USA). The percentage of cell viability was calculated as follows: [(OD 570 nm of experimental well)/(OD 570 nm of control well)] × 100%. Our cell viability assay experiment was approved by the Committee on Biological Research of National Taiwan Normal University and was implemented under the guidelines of the committee.

### 2.3. Animal Preparation

All ICR mice were purchased from BioLASCO Taiwan Yi-Lan Breeding Center, which has been awarded the Full Accreditation of AAALAC International. Our animal experiments were approved by the Institutional Animal Care and Use Committee of National Taiwan Normal University (Protocol number: NTNU Animal Experiments No. 104002). In accordance with the Institutional Guidelines of the Animal Care and Use Committee of NTNU, ICR mice were maintained in the animal facility at NTNU under specific pathogen-free conditions. A total of 12 female ICR mice were randomised into four groups: wild-type mice with sham treatment (WT + sham), wild-type mice with B401 treatment (WT + B401), bilateral ovariectomised mice with sham treatment (OVX + sham), and bilateral ovariectomised mice with B401 treatment (OVX + B401). All mice were housed in a constant temperature environment at 22°C ± 2°C with a 12-h light/dark cycle, and mice had* ad libitum* access to water and food. Mice received oral herbal formula B401 treatment at a dose of 30 mg/kg bw/day for 2 weeks.

### 2.4. Blood Biochemical Analysis

Levels of calcium and phosphorus were assayed using colorimetric assay kits (Biovision, Milpitas, CA, USA), and the concentration of estradiol-17*β* was assayed using an Assay Design EIA kit (Assay Design, Ann Arbor, MI, USA). Measurements of blood calcium and phosphorus levels were manipulated in line with the manufacturer's protocol. In addition, levels of ROS in the blood of the four groups of ICR mice were determined using lucigenin- and luminol-amplified chemiluminescence (CL) methods to measure O2^•−^ and H_2_O_2_ activity. Blood levels of ROS were measured using a CL analyzer (CLA-ID3 CL analyzer; Tohoku Electronic Industrial Co., Ltd., Sendai, Japan) after 1.0 mL of 0.1 mM lucigenin in phosphate-buffered saline (PBS, pH 7.4) was added to the blood samples of mice. The total CL counts of the four groups were calculated by integrating the area under the curve within 600 s.

### 2.5. Immunohistochemistry

After herbal formula B401 or sham treatment, the four groups of ICR mice were anaesthetised with urethane (1.5 mg/kg); subsequently, cardiac perfusion with PBS containing 4% formaldehyde (Sigma-Aldrich Corporation) was conducted. Uterine tissue specimens of mice were obtained and fixed with 4% formaldehyde (EM grade) (Sigma-Aldrich Corporation). The uterine tissue specimens were embedded in paraffin and then cut into 5-*μ*m-thick sections. Some sections were mounted on slides and then assessed through hematoxylin and eosin (H&E) staining with a kit-based approach (Sigma-Aldrich Corporation). For immunohistochemistry (IHC) staining, following the heat-induced epitope retrieval method, uterine tissue sections were separately stained at room temperature for 1 h with antibodies for estrogen receptor *α* (ER*α*), estrogen receptor *β* (ER*β*), progesterone receptor (PR), and vascular endothelial growth factor (VEGF) (Cell Signaling Technology Inc., Danvers, MA, USA). Then, immunostaining was detected through incubation with biotinylated secondary antibodies (Novolink™ Polymer Detection System l, Leica Biosystems Newcastle Ltd., Newcastle, UK) for 30 min and the avidin–biotin–horseradish peroxidase (HRP) complex (Novolink™ Polymer Detection System l, Leica Biosystems Newcastle Ltd.) for additional 30 min. Immunostaining was visualised using DAB Chromogen (Novolink™ Polymer Detection System l, Leica Biosystems Newcastle Ltd.), and the slides were counterstained with hematoxylin (Novolink™ Polymer Detection System l, Leica Biosystems Newcastle Ltd.).

### 2.6. Western Blot Analysis

After herbal formula B401 or sham treatment, the four groups of ICR mice were anaesthetised with urethane (1.5 mg/kg); subsequently, transcardial perfusion with physiological saline was conducted. Uterine tissue specimens were homogenised in a buffer solution. Then, proteins in the separated solution were quantified using a BCA protein assay kit (Thermo Fisher Scientific Inc., Waltham, MA, USA) and separated on 12.5% or 15% SDS polyacrylamide gels (Bionovas Pharmaceuticals Inc., Washington DC, USA). The separated proteins were transferred to polyvinylidene difluoride membranes (GE Healthcare Life Sciences, Barrington, IL, USA). The antibodies used in this study were *β*-actin (Thermo Fisher Scientific Inc.) and superoxide dismutase 2 (SOD2) (Cell Signaling Technology Inc.), which were detected using suitable HRP-conjugated secondary antibody (Santa Cruz Biotechnology Inc.). Immunostaining was visualised using the enhanced chemiluminescence (ECL) substrate (Millipore, Billerica, MA, USA) and was quantified using ImageJ analysis software (version 1.48t, NIH, Wayne Rasband, Washington DC, USA).

### 2.7. Statistical Analysis

All data are presented as mean ± SEM. The data were analysed using one-way or two-way ANOVA followed by Student–Newman–Keuls multiple comparison post-test. A *P* value of less than 0.05 was considered significant.

## 3. Results

### 3.1. Chromatographic Fingerprint of the Herbal Formula B401

**Figure 1:**
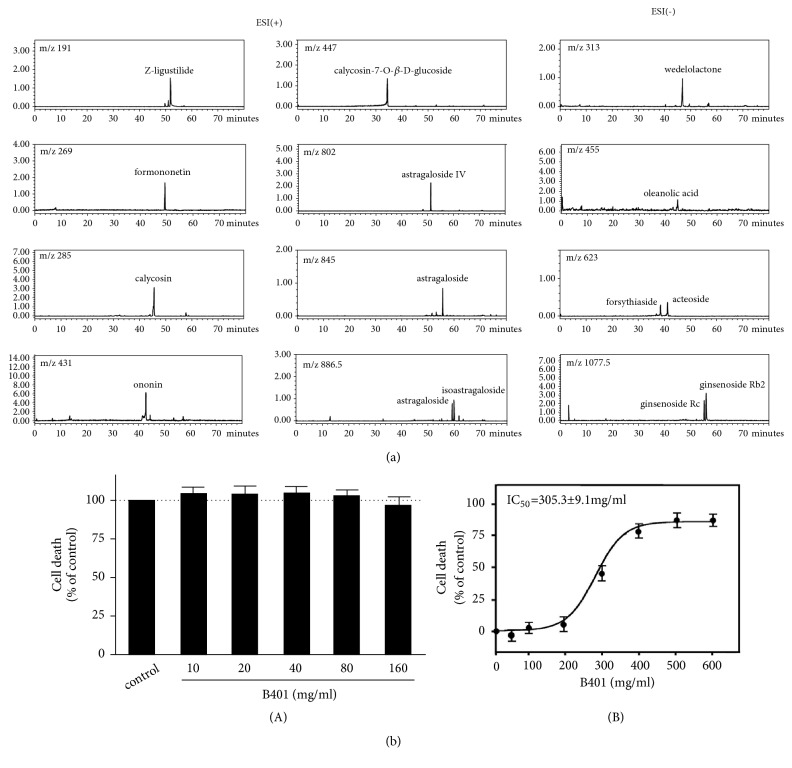
Chromatographic fingerprint analysis and cytotoxicity assay of the Chinese herbal formula B401. (a) Chromatographic fingerprint analysis was conducted through the LC/MS. Fifteen bioactive marker substances from the components of the herbal formula B401 were qualitatively determined within 80 min under selected LC/MS conditions. (b) (A) Cell viability of herbal formula B401 treatment measured through the MTT assay of SH-SY5Y cells. (B) The IC_50_ values of the Chinese herbal formula B401 in SH-SY5Y cells were calculated using the dose–response curve. The results are shown as mean ± SEM, and experiments were repeated three times for each treatment.

Figure 1(a) shows the chromatographic fingerprint of the herbal formula B401 obtained using LC/MS. Fifteen bioactive marker substances from the components of the herbal formula B401 were qualitatively measured using LC/MS under selected conditions. These bioactive marker substances included Z-ligustilide from* Ang. sinensis*; astragaloside, isoastragaloside, astragaloside, and astragaloside IV from* Ast. membranaceus*; calycosin-7-O-*β*-D-glucoside, ononin, and wedelolactone from* E. prostrata*; oleanolic acid from* L. fructus*; ginsenoside Rc and ginsenoside Rb2 from* P. ginseng*; and calycosin, formononetin, forsythiaside, and acteoside from* R. glutinosa*.

### 3.2. IC_*50*_ Values of the Herbal Formula B401

Figure 1(b)(A) shows the potential cytotoxic effect of the herbal formula B401, which was measured through the MTT assay of SH-SY5Y cells. Cells were treated with the herbal formula B401 at the concentrations of 10, 20, 40, 80, and 160 mg/mL. After 24-h B401 treatment, cell viability was examined using the MTT assay. The herbal formula B401 showed no significant cytotoxicity at any concentration. Figure 1(b)(B) shows the dose–response sigmoid curve of the herbal formula B401. The calculated IC_50_ value of the herbal formula B401 was 305.3 mg/mL for SH-SY5Y cells.

### 3.3. Oral B401 Treatment Effectively Increases Levels of Calcium, Phosphorus, and Estradiol-17*β* in the Blood of Ovariectomised Mice

**Figure 2:**
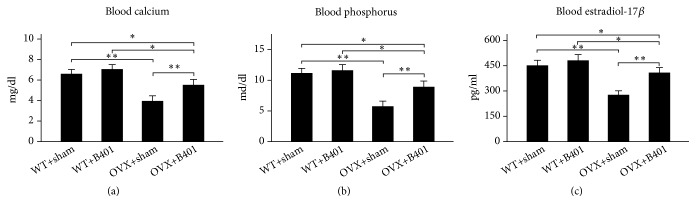
Oral B401 treatment effectively increases blood levels of calcium and phosphorus and serum levels of estradiol-17*β* in ovariectomized mice. Statistical comparison of quantified levels of (a) calcium, (b) phosphorus, and (c) estradiol-17*β* in blood/serum among WT + sham, WT + B401, OVX + sham, and OVX + B401 mice. The results are shown as mean ± SEM (*∗∗P* < 0.01, *∗P* < 0.05, two-way ANOVA followed by Student–Newman–Keuls multiple comparison post-test), and experiments were repeated three times for each treatment.

Figures 2(a) and 2(b) illustrate quantified blood levels of calcium and phosphorus in WT + sham, WT + B401, OVX + sham, and OVX + B401 mice. Quantified blood levels of calcium and phosphorus in OVX + sham mice significantly decreased compared with those in WT + sham mice (*P* < 0.01), whereas quantified blood levels of calcium and phosphorus in OVX + B401 mice significantly increased compared with those in OVX + sham mice (*P *< 0.01). Figure 2(c) presents quantified serum concentrations of estradiol-17*β* in WT + sham, WT + B401, OVX + sham, and OVX + B401 mice. Quantified serum concentrations of estradiol-17*β* in OVX + sham mice significantly decreased compared with those in WT + sham mice (*P* < 0.01), whereas quantified serum concentrations of estradiol-17*β* in OVX + B401 mice significantly increased compared with those in OVX + sham mice (*P* < 0.01).

### 3.4. Oral B401 Treatment Effectively Increases Uterine Weight in Ovariectomised Mice

**Figure 3:**
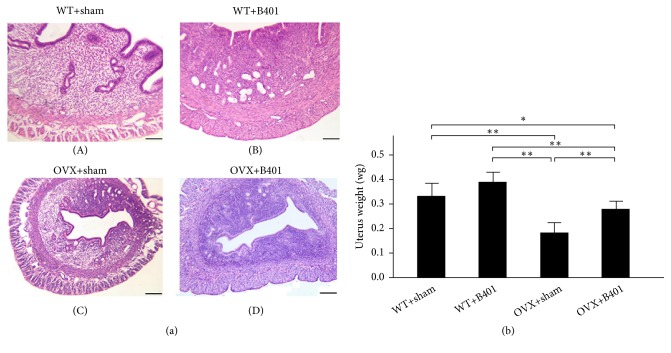
Oral B401 treatment effectively increases uterine weight in ovariectomized mice. (a) Uterine morphology in WT + sham, WT + B401, OVX + sham, and OVX + B401 mice through H&E staining. Bar scale = 100 *μ*m. (b) Statistical comparison of quantified uterine weight among WT + sham, WT + B401, OVX + sham, and OVX + B401 mice. The results are shown as mean ± SEM (*∗∗P* < 0.01, *∗P* < 0.05, two-way ANOVA followed by Student–Newman–Keuls multiple comparison post-test), and experiments were repeated three times for each treatment.

As shown in Figure 3(a), uterine morphology in WT + sham, WT + B401, OVX + sham, and OVX + B401 mice was observed and then compared. The thickness of the uterine wall in OVX + sham mice decreased compared with that in WT + sham mice, whereas the thickness of the uterine wall in OVX + B401 mice increased compared with that in OVX + sham mice.  Figure 3(b) shows that the quantified uterine weight of OVX + B401 mice significantly decreased compared with that of WT + sham mice (*P* < 0.01), whereas the quantified uterine weight of OVX + B401 mice significantly increased compared with that of OVX + sham mice (*P* < 0.01).

### 3.5. Oral B401 Treatment Increases Expression of the VEGF, ER*α*, ER*β*, and PR in the Uterine Tissue of Ovariectomised Mice

**Figure 4:**
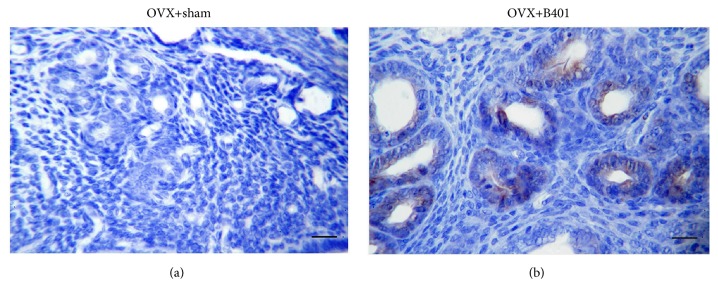
Oral B401 treatment enhances expression of the vascular endothelial growth factor (VEGF) in the uterine tissue of ovariectomised mice. Uterine VEGF expressions (expressed by deep brown color) in (a) OVX + sham and (b) OVX + B401 mice through IHC staining. Bar scale = 20 *μ*m.

**Figure 5:**
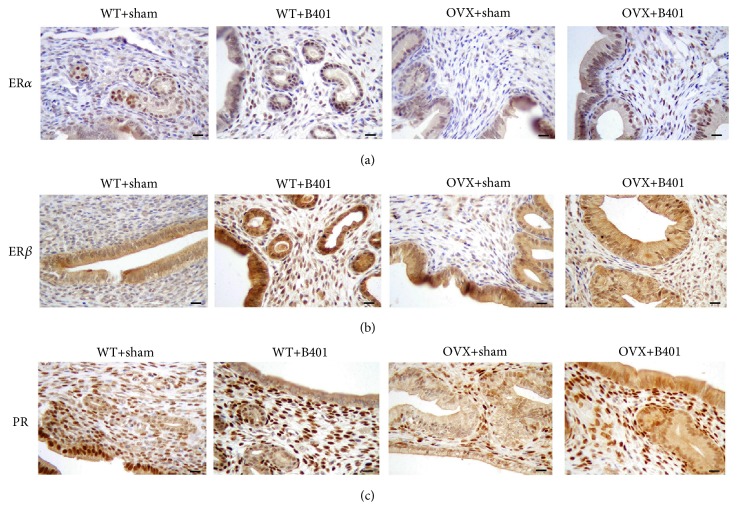
Oral B401 treatment enhances expressions of the estrogen receptor *α* (ER*α*), estrogen receptor *β* (ER*β*), and progesterone receptor PR in the uterine tissue of ovariectomised mice. Uterine expression of (a) ER*α*, (b) ER*β*, and (c) PR (expressed by deep brown color) in WT + sham, WT + B401, OVX + sham, and OVX + B401 mice through IHC staining. Bar scale = 20 *μ*m.

In this study, we examined the expression of VEGF in the uterine tissue in the OVX + sham and OVX + B401 mice (Figure 4). We found that OVX + B401 mice showed higher expression of VEGF in the uterine tissue than OVX + sham mice. In addition, we examined the expression of ER*α*, ER*β*, and PR in the uterine tissue in the four groups (Figure 5). We found that OVX + sham mice showed lower expression of ER*α*, ER*β*, and PR in the uterine tissue than WT + sham mice, whereas OVX + B401 mice showed higher expression of ER*α*, ER*β*, and PR in the uterine tissue than OVX + sham mice.

### 3.6. Oral B401 Treatment Effectively Reduces Blood ROS and Increases Uterine SOD2 Expression in Ovariectomised Mice

**Figure 6:**
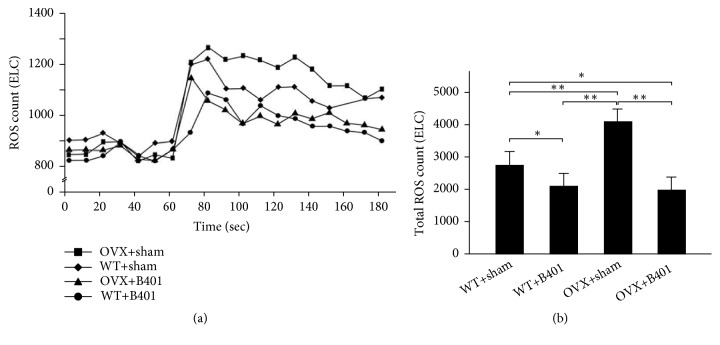
Oral B401 treatment effectively reduces blood ROS in ovariectomised mice. (a) Blood ROS count in WT + sham, WT + B401, OVX + sham, and OVX + B401 mice through chemiluminescence analysis. (b) Statistical comparison of quantified ROS count among WT + sham, WT + B401, OVX + sham, and OVX + B401 mice. The results are shown as mean ± SEM (*∗∗P* < 0.01, *∗P*< 0.05, two-way ANOVA followed by Student–Newman–Keuls multiple comparison post-test), and experiments were repeated three times for each treatment.

**Figure 7:**
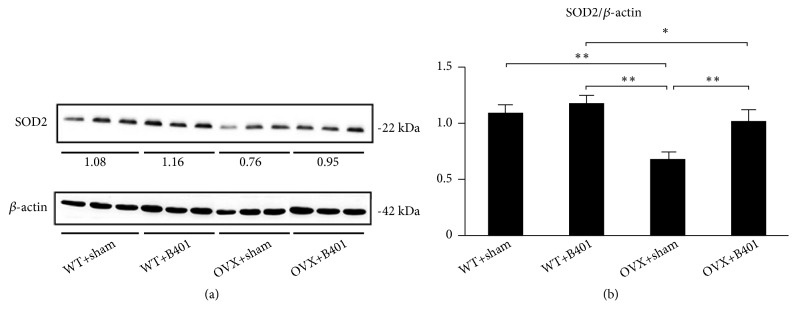
Oral B401 treatment effectively enhances SOD2 expression in the uterine tissue of ovariectomised mice. (a) Uterine SOD2 expression levels in WT + sham, WT + B401, OVX + sham, and OVX + B401 mice through Western blotting. (b) Statistical comparison of quantified SOD2 expression among WT + sham, WT + B401, OVX + sham, and OVX + B401 mice. The results are shown as mean ± SEM (*∗∗P*<0.01, *∗P*< 0.05, two-way ANOVA followed by Student–Newman–Keuls multiple comparison post-test), and experiments were repeated three times for each treatment.

To study the effects of the herbal formula B401 on mitochondrial oxidative stress in ovariectomised mice, we examined and then compared the blood ROS count and uterine SOD2 expression among the four groups (Figures 6 and 7).  Figure 6(a) shows that OVX + sham mice showed higher blood ROS counts than WT + sham mice, whereas OVX + B401 mice showed lower blood ROS counts than OVX + sham mice.  Figure 6(b) presents that quantified ROS counts in the blood of OVX + sham mice significantly increased compared with the counts in the blood of WT + sham mice (*P* < 0.01), whereas quantified ROS counts in the blood of OVX + B401 mice significantly decreased compared with the counts in the blood of OVX + sham mice (*P *< 0.01). SOD2 is an antioxidant enzyme for oxidative stress.  Figure 7(a) shows uterine SOD2 expression levels in WT + sham, WT + B401, OVX + sham, and OVX + B401 mice. As presented in Figure 7(b), quantified uterine SOD2 expression was significantly decreased in OVX + sham mice than in WT + sham mice (*P* < 0.01), whereas quantified uterine SOD2 expression was significantly increased in OVX + B401 mice than in OVX + sham mice (*P* < 0.01).

## 4. Discussion

### 4.1. Reproductive Regulation of the Herbal Formula B401 in Ovariectomised Mice

In our previous studies, the herbal formula B401 treatment should be viable alternative treatment for the management of neurodegenerative diseases such as Alzheimer's, Huntington's, and Parkinson's diseases [9–11, 13]. In addition, we observed that most menopausal women who took the herbal formula B401 also felt that sweating, anxiety, and sleep disorders were significantly relieved. Therefore, the herbal formula B401 is currently used as a health food for middle-aged women in Taiwan. Before studying reproductive regulation of the herbal formula B401 in the ovariectomised mouse model, we should reexamine the active ingredients and cytotoxicity of the herbal formula B401. According to the MTT assay, the calculated IC_50_ value of the herbal formula B401 was 305.3 mg/mL for SH-SY5Y cells (Figure 1(b)). The IC_50_ dose of SH-SY5Y cells converted to the IC_50_ dose of ovariectomised mice should be 305 g/Kg bw/day. If safety factor calculated from 1/10 as the starting dose of human, the safety dose of the herbal formula B401 for an adult woman weighing 60 kilograms should be 1,830 g per day. In this study, those ovariectomised ICR mice received oral herbal formula B401 treatment at a dose of 30 mg/kg bw/day for 2 weeks. The converted edible dose of the herbal formula B401 for an adult woman weighing 60 kilograms is 1.8 g per day that should be much smaller than the dose of IC_50_ value of the herbal formula B401. Thus, the herbal formula B401 should be quite safe dosage for menopausal women.

Ovariectomised female rats have a higher risk of osteoporosis [14]. In a previous study, bone mineral (calcium and phosphorus) contents were decreased in postmenopausal ovariectomised female rats, and estrogen therapy could reduce postmenopausal bone loss [15]. In addition, Estradiol-17 beta therapy could enhance endosteal bone formation in the ovariectomized mice [16]. These results clearly support our study results that ovariectomised female mice showed significant decrease in levels of calcium, phosphorus, and estradiol-17*β* in the blood, while showing significant increased levels of calcium, phosphorus, and estradiol-17*β*in the blood after the herbal formula B401 therapy (Figure 2).

It has been reported that the reduction of uterine weight in ovariectomised rats might be due to reduced uterine epithelial height, expanded uterine stroma, reduced uterine myometrial thickness, and atrophic vaginal epithelium [17, 18]. In this study, we observed that ovariectomised female mice showed endometrium atrophy because of uterine weight reduction, while the herbal formula B401 therapy could increase uterine weight and endometrial thickness (Figure 3). This amelioration of uterine atrophy in ovariectomised mice might be probably due to the presence of biologically active phytoestrogen-like compounds in the herbal formula B401. Furthermore, through IHC, we found that the herbal formula B401 therapy could enhance the expression of VEGF in the uterine tissue of ovariectomised rats (Figure 4). Similar results have also been reported that ovariectomised rats with estradiol-17*β* therapy can increase endometrial blood flow and reduced endometrial vascular resistance [19]. The increased endometrial blood flow in ovariectomised rats with estradiol-17 beta treatment is due to increasing nitric oxide synthesis and subsequently improving endothelial function [20].

It has been reported that estrogen may play multiple biological functions by modulating ER*α* and ER*β* [2], and PR expression can be induced by estrogen [21]. Thus, we also examined the expression of ER*α*, ER*β*, and PR in the uterine tissue. In this study, ovariectomised mice showed significantly decreased levels of estradiol-17*β* in the blood. We further found that ovariectomised mice significantly decrease expressions of uterine ER*α*, ER*β*, and PR, whereas the herbal formula B401 therapy could enhance expressions of uterine ER*α*, ER*β*, and PR (Figure 5). These results clearly indicate that the herbal formula B401 can increase estradiol-17*β* levels and increase the expression of ER*α*, ER*β*, and PR in the uterine tissue. It is reasonable that the herbal formula B401 can be an alternative treatment for reproductive regulation.

### 4.2. Oxidative Stress Alleviation of the Herbal Formula B401 in Ovariectomised Mice

It has been reported that menopause is associated with metabolic disorders and oxidative stress [14]. A previous study demonstrated that ovariectomised rats showed enhanced oxidative stress in the plasma. Moreover, it has been reported that estrogen can produce antioxidant effects and reduce blood ROS [22, 23]. It has been reported that ROS can cause oxidative damage to cells, while SOD2 is an antioxidant enzyme that is responsible for O^2−^ dismutation to H_2_O_2_ [24]. Reduced SOD activity in ovariectomised animals may induce more accumulation of O^2−^ and inhibit other antioxidant enzymes [25]. The enhancement of the blood ROS in ovariectomised mice clearly suggests that oxidative stress is the major factor underlying menopausal symptoms. In this study, we evaluated the beneficial effects of the herbal formula B401 on mitochondrial oxidative stress in ovariectomised mice. Our study showed that ovariectomised mice significantly increase blood ROS, while the herbal formula B401 therapy could significantly decrease blood ROS (Figure 6). Furthermore, the herbal formula B401 therapy could significantly enhance uterine SOD2 expressions (Figure 7). Thus, we suggested that the herbal formula B401 might be an alternative treatment for mitochondrial oxidative stress alleviation in the reproductive regulation.

## 5. Conclusions

The herbal formula B401 therapy can enhance levels of calcium, phosphorus, and estradiol-17*β* in the blood, increase uterine weight and endometrial thickness, and improve expressions of ER*α*, ER*β*, PR, and SOD2 in the uterine tissue but decrease blood ROS levels in ovariectomised mice. Thus we suggested that the herbal formula B401 can be a health supplement for alleviating discomfort symptoms in middle-aged women.

## Data Availability

The subcutaneous blood flow assay, blood biochemical analysis, ROS analysis, immunohistochemistry, and Western blot analysis data used to support the findings of this study are included within the article and available from the corresponding author upon request.

## Abbreviations

ER*α*: Estrogen receptor *α*
ER*β*: Estrogen receptor *β*
IC_50_: Half maximal inhibitory concentration
IHC: Immunohistochemistry
LC/MS: Liquid chromatography/mass spectrometry
MTT: 3-(4,5-Dimethylthiazol-2-yl)- 2,5-diphenyltetrazolium bromide
OVX: Ovariectomized
PR: Progesterone receptor
ROS: Reactive oxygen species
SEM: Standard error of the mean
SOD2: Superoxide dismutase 2
WT: Wild-type.
